# Evaluation of the Use of Sub-Immunodominant Antigens of *Babesia bovis* with Flagellin C Adjuvant in Subunit Vaccine Development

**DOI:** 10.3390/vaccines12111215

**Published:** 2024-10-25

**Authors:** Manuel J. Rojas, Reginaldo G. Bastos, Jinna A. Navas, Heba F. Alzan, Jacob M. Laughery, Paul A. Lacy, Massaro W. Ueti, Carlos E. Suarez

**Affiliations:** 1 Department of Veterinary Microbiology & Pathology, College of Veterinary Medicine, Washington State University, Pullman, WA 99164, USA; mjrojas@wsu.edu (M.J.R.); reginaldo.bastos@usda.gov (R.G.B.); jnavas@wsu.edu (J.A.N.); heba.alzan@wsu.edu (H.F.A.); j.laughery@wsu.edu (J.M.L.); massaro.ueti@usda.gov (M.W.U.); 2 Animal Health Department, Universidad Nacional de Colombia, Bogota 111321, Colombia; 3 Animal Disease Research Unit, Agricultural Research Service, US Department of Agriculture, Pullman, WA 99164, USA; lacyp@wsu.edu; 4 Parasitology and Animal Diseases Department, National Research Center, Giza 12622, Egypt

**Keywords:** *Babesia bovis*, babesiosis, epitopes, vaccines, RAP-1, RRA

## Abstract

Bovine babesiosis caused by the tick-borne apicomplexan parasite *Babesia bovis* remains a threat for cattle worldwide, and new vaccines are needed. We propose using immune-subdominant (ISD) antigens as alternative vaccine candidates. We first determined that RAP-1 NT and RRA are subdominant antigens using recombinant antigens in ELISAs against sera from *B. bovis*-protected cattle. Protected animals demonstrated high antibody responses against the known immunodominant rRAP-1 CT antigen, but significantly lower levels against the rRAP-1 NT and rRRA antigens. Next, a group of cattle (n = 6) was vaccinated with rRRA and rRAP-1 NT using a FliC–Emulsigen mix as the adjuvant, and there was a control group (n = 6) with the adjuvant mix alone. All but one immunized animal demonstrated elicitation of strong humoral immune responses against the two ISD antigens. Acute babesiosis occurred in both groups of cattle upon a challenge with the virulent *B. bovis*, but a significant delay in the average rate of decrease in hematocrit in the vaccinated group, and an early monocyte response, was found in half of the vaccinated animals. In conclusion, we confirmed the immune subdominance of rRRA and rRAP-1 NT and the ability of FliC to increase immunogenicity of ISD antigens and generate useful information toward developing future subunit vaccines against *B. bovis*.

## 1. Introduction

*Babesia bovis* is a tick-borne apicomplexan parasite responsible for acute and persistent bovine babesiosis [1,2]. The disease remains an important limiting factor for cattle production in tropical and semitropical regions worldwide. Current approaches for prevention and control remain inadequate, and improvements are urgently needed [3]. Live vaccines are effective to prevent acute disease, but they can only be applied to young calves and may risk unknown impurities from vaccine donor blood, require a cold chain, have the risk of reversion to virulence, and can be costly to produce and distribute, among other important limitations [1,3]. Other promising options toward improved control also include the possible development use of transmission-blocking vaccines using a recombinant protein representing a sexual-stage antigen [4]. However, blood-stage subunit vaccines would also be ideal, but so far, most, if not all reported vaccine trials developed in cattle using vaccines based on recombinant proteins failed in providing acceptable levels of protection [3]. These failures could be in part attributed to the existence of numerous gaps in knowledge, including the nature and characteristics of protective antigens, the identification of appropriate adjuvants, and a better understanding of the immune mechanisms that are involved in protective immune responses.

Similar to other members of the order piroplasmida, *Babesia* parasites encode a family of *rap-1* genes [5,6,7]. The *rap-1* gene family *B. bovis* includes two identical canonical *rap-1* genes and a single gene copy of the RAP-1-related antigen (*rra*) gene, both located in chromosome IV and separated by a ~90 kb intergenic region [8,9]. The *rap-1* genes are syntenic and conserved among the *Theileria*, *Cytauxon*, and *Babesia* parasites, but the *rra* genes likely evolved after *Babesia* speciation and are only found in all *Babesia* genomes analyzed so far. If this association is further demonstrated, the presence of an *rra* gene may become a hallmark of *Babesia* spp, which can also be used to discriminate sensu stricto from sensu lato *Babesias*-like organisms [10]. The canonical Babesial *rap-1* and the *rra* genes encode for a relatively well-conserved region of 310 amino acids in their NT end. The RAP-1-defining motifs of the NT domain of the *B. bovis* rhoptry-associated protein-1 (RAP-1) include an arrangement of four conserved cysteine residues, a well-conserved 14 amino acid sequence and its equivalent 15-mer of RAP-1-related antigen (RRA) [11], and a series of conserved short motifs [11,12]. It was also established previously that the sequence-defined domains of RAP-1 have a structural correlate. Modeling reveals conserved globin-like structures present in all RAP-1 and RRA in piroplasmid parasites, as well as in other RAP-1-like molecules present in other organisms [11,13]. The remarkable level of structural conservation found among species, even in the face of very low sequence identity, suggests that these are functionally essential proteins in apicomplexan parasites, albeit of unknown function, and thus potential targets for control measures [9,11]. Altogether, sequence conservation and the structural findings suggest that the main function of the RAP-1 molecules resides in its well structurally conserved NT region [11]. In contrast to RRA, RAP-1 also contains an immunodominant CT region with a previously defined B-cell epitope that maps to a region of 23 amino acid repeats [14]. The RAP-1 CT domain contains the remaining 255 amino acid residues of the *B. bovis* RAP-1 protein, does not contain sequences that are conserved in all Babesial RAP-1, and is essentially made of a series of highly antigenic degenerate 23 amino acid repeats [14]. Furthermore, structural analysis also predicted a poorly species-conserved structure for the *B. bovis* RAP-1 CT region [13]. The presence of this repeat-rich CT region likely makes the *B. bovis* RAP-1 an immunodominant molecule [14]. Thus, in summary, and in contrast to the well structurally conserved RAP-1 NT region, the CT region lacks the Babesial RAP-1 species-conserved sequences and has structural features that are also not conserved among other apicomplexan parasites.

It has been proposed that immune-subdominant antigens may prove to be more effective as *Babesia* vaccine candidates than immunodominant antigens [15]. This appears as a valid criterion for selecting vaccine candidates, considering that *Babesia* parasites are able to persist in their hosts causing long-term chronic infections in the face of strong immune responses. It is reasonable to argue that sequences in essential molecules were selected as a part of a co-evolutionary process, because targeting them by protective immune responses would be, at least in theory, lethal for the parasite. Such immune-subdominant antigens that do not naturally evoke strong immune responses are thus possible valid candidates for vaccine development, especially if they are exposed to the effectors of the immune system and if their immunogenicity can be augmented using certain adjuvants.

An interesting observation transpiring from previous antigenic, sequence, and structural analyses is that RRA can be considered as a truncated version of RAP-1 which lacks a region with highly antigenic repeats in its CT end and was characterized as a neutralization-sensitive, albeit immune-subdominant (ISD) antigen [9], but to our knowledge, was never tested as an antigen in vaccination trials. Furthermore, previous data are consistent with the notion that, similar to RRA and in contrast to its CT region, the well-conserved NT end region of RAP-1 is also immune-subdominant [11]. Consistently, we recently demonstrated the presence of at least one immune-subdominant B-cell epitope in the well-conserved 15-mer motif of *B. bovis* RRA, which elicits a weak antibody response in vaccinated and challenged cattle. This is in contrast to strong antibody responses that these animals elicited against a B-cell epitope that is present in one of the repeats of the CT region of RAP-1 [11].

It is possible that RAP-1 and RRA share similar functions, but it is also conceivable that the robust immune responses elicited by the full-size RAP-1 molecules drove the evolution and selection of parasites that can also express a subdominant and poorly expressed version of the protein. Furthermore, it is rational to speculate that RRA may take over RAP-1 functions in the face of neutralizing immune responses against the canonical RAP-1. In this scenario, the possible interplay between these two proteins may also have implications for the development of persistent infections of cattle by *B. bovis*, where the parasite can survive in the bovine host despite the presence of high antibody levels against essential or functionally relevant proteins.

An important gap limiting rational subunit vaccine design includes the selection of an optimal adjuvant. Most, if not all, previously reported *Babesia* vaccine trials involving recombinant proteins were performed using classic adjuvants such as saponin and its derivatives [16]. Although saponin adjuvants proved to be effective at promoting high levels of antibodies, none of the previously reported trials resulted in achieving satisfactory levels of protection [16], suggesting that alternative approaches, including the use of immunostimulant molecules, need to be tested. Flagellin C or flagellar filament structural protein (FliC) adjuvants are known to be able to stimulate the TLR5 receptor, resulting in activation of innate immune responses, including the production of NO, a known effector against *B. bovis* [17]. Furthermore, early activation of innate immune responses is a known essential feature in protective immune mechanisms against *Babesia* parasites. Thus, adjuvants such as FliC that can stimulate the type of immune responses that are known to be needed to elicit protection against *Babesia* parasites are optimal candidates to test in novel subunit vaccines [17]. Importantly, the identification of two sub-immunodominant antigens would also provide an optimal experimental model for testing the potency of FliC as a possible adjuvant in cattle, as well as its ability to potentiate immunogenicity of an sub-immunodominant antigen in a classic immunization trial.

The objectives of this study were, first, to confirm the sub-immunodominance of RRA and RAP-1 NT by determining levels of antibodies in cattle vaccinated with a live *B. bovis* vaccine and subsequently challenged with virulent parasites. This would provide a source of sub-immunodominant antigens to test if such antigens can elicit high antibody responses when administrated with FliC adjuvant in an experimental vaccine and if such a vaccine, based on sub-immunodominant antigens, can elicit immune responses able to help the bovine host to survive the devastating effects of acute infection caused by virulent strains of *B. bovis*.

## 2. Materials and Methods

### 2.1. Parasites and Sera from B. bovis-Protected Cattle

The crude antigen mix used in the immunoblot analysis was obtained from in vitro-cultured parasites of the Mo7 biological clone of *B. bovis* as described previously [18]. The *B. bovis* parasites were grown in long-term microaerophilic stationary-phase culture [18].

Pre-immune and immune sera from the *B. bovis* experimentally infected cattle used in the iELISA analysis described in Section 3.1 were generated in previous studies [19,20].

### 2.2. Recombinant Proteins and Immunoblot Analysis

The production, purification, and testing of the *B. bovis* RRA (XP_001610950.1), RAP-1 CT, and RAP-1 NT recombinant proteins, derived from vector pET-30a(+) recombinant plasmids, were reported previously [11]. All experimental procedures used in the immunoblots were as described previously [11]. The purity and specificity analyses of the recombinant proteins using Coomassie blue staining and immunoblots [11] are depicted in Supplementary Figure S1.

### 2.3. Serological Analysis

Serological analysis for the detection of antibodies against the *B. bovis* recombinant RRA, RAP-1 NT, and RAP-1 CT proteins was performed using indirect ELISA (iELISA) methods [11], using sera from vaccinated and control animals. Optical density values (OD) are presented as the average and standard deviations of technical replicates. ANOVA test was performed for statistical analysis, and *p*-value < 0.05 was considered as a significant difference.

### 2.4. Study Design

A group of 6 eight-month-old, *Babesia*-free Holstein calves were vaccinated subcutaneously with a mix of recombinant RRA and RAP-1 NT proteins (50 μg per inoculation) plus adjuvant. Another group with 6 animals was injected with the adjuvant mix alone and referred to henceforth as the mock-vaccinated group. The adjuvant used was a mix composed of rFliC (30 μg) and Emulsigen^®^ (200 μL), (MVP Technologies, Cooper City, FL, USA). Emulsigen^®^ was included in the adjuvant mix in order to provide slow release of antigens for continuing immune stimulation. Using 12 calves in this experiment provided a power = 90% and Alpha = 0.05 as determined using the Sample Size Calculator (clincalc.com). Following 4 subcutaneous immunizations (at 15-day intervals), vaccinated and mock-vaccinated animals were challenged intravenously with stabilate containing 10^7^ infected red blood cells (iRBC) of the virulent homologous *B. bovis* strain Vir-S74-T3Bo. Previous studies have shown that a challenge dose of 10^7^ iRBC of Vir-S74-T3Bo induces severe acute disease in cows with the onset of clinical signs starting approximately 9–10 days after infection [20]. All animals were monitored daily after the challenge for clinical signs of acute bovine babesiosis. The parameters measured included fever, drop in hematocrit (Hct), and presence of parasites in peripheral blood. Animals were also checked for other clinical manifestations of acute babesiosis such as anorexia, prostration, and neurological signs. Statistical comparisons of averaged data obtained from temperature and hematocrit measurements, performed at several timepoints after vaccination and challenge, were performed with a two-tailed *t*-test using GraphPad Prism software version 9 (GraphPad Software, San Diego, CA, USA). A *p*-value < 0.05 was considered statistically significant.

Humoral immune responses in vaccinated, mock-vaccinated, and challenged cattle were evaluated by iELISA using recombinant RRA and RAP-1 NT proteins and control proteins, as described above.

### 2.5. Assesment of Parasite Load in Peripheral Blood

*B. bovis* load in peripheral blood of animals after vaccination and challenge was assessed by real-time quantitative PCR (qPCR), as described previously [20,21]. To this end, blood from all 12 experimental animals was collected via jugular venipuncture using Vacutainer^®^ tubes containing ethylenediamine tetra acetic acid (EDTA) (BD Company, Franklin Lakes, NJ, USA) on days 1, 3, 5, and 6 after challenge. Genomic DNA from whole blood, extracted using the QIAamp^®^ DNA Blood Mini Kit (QIAGen, Valencia, CA, USA), was used for qPCR to amplify the single-copy *B. bovis* msa-1 gene. The specific primers used were (5′ gatgcgtttgcacatgctaag 3′ and 5′ cgggtacttcggtgctctca 3′). The sequence of the probe used in the qPCR was (FAM 5′-cacgctcaagtaggaaattttgttaaacctgga-3′ TAMRA). The qPCR reactions were performed at 95 °C for 2 min, 40 cycles of 95 °C for 15 s, 55.8 °C for 45 s, and extension at 72 °C for 1 min. A *msa-1* standard curve was prepared with 10^0^ to 10^7^ plasmid copies, and all samples were run in technical triplicate, as described elsewhere [20]. The results of parasite load in peripheral blood are presented as the Quantification Cycle (Cq) of the qPCR, as an indirect measurement of parasitemia [21,22]. Average results of parasite load in vaccinated and control animals were compared by a two-tailed *t*-test. A *p*-value <0.05 was considered statistically significant.

### 2.6. Hematological Analysis

Experimental animals were evaluated at several timepoints using complete cell blood counts after vaccination and challenge. Hematological counts were performed using the ProCyte One™ Hematology Analyzer (IDEXX Laboratories, Westbrook, ME, USA). The analyzed samples consisted of peripheral blood obtained from all experimental animals collected in Vacutainer^®^ tubes containing EDTA at several timepoints after vaccination and challenge. The numbers of total leukocytes, lymphocytes, monocytes, neutrophils, and red blood cells (RBCs) were measured from blood samples that were homogenized for 5 min. Results of each leukocyte population are presented as 1000 cells/μL of blood, and RBCs are shown as hematocrit percentage (Hct %). The averaged results of cell count in all experimental animals, as well as the serological results, were compared by a two-tailed *t*-test and ANOVA, and a *p*-value <0.05 was considered statistically significant. For all the data, the statistics program performed variance checks by means of Levene’s test as a way to confirm normal distribution of the data.

For the calculation of the hematocrit decay slopes, we used the following formula:

y = m x + b

where “m” is the calculated slope for each curve, “x” represents time domain (days post-challenge, DPC), and “y” represents percentage of RBCs (Hct %). Additionally, to find the intersection of the slope and the 50% of the initial Hct, we used the formula x = y − b/m, where x represents the day when each group would reach the critical 50% Hct.

## 3. Results

### 3.1. Recognition of rRRA and rRAP-1 NT by Antibodies in Sera of B. bovis-Immunized Cattle

**Figure 1:**
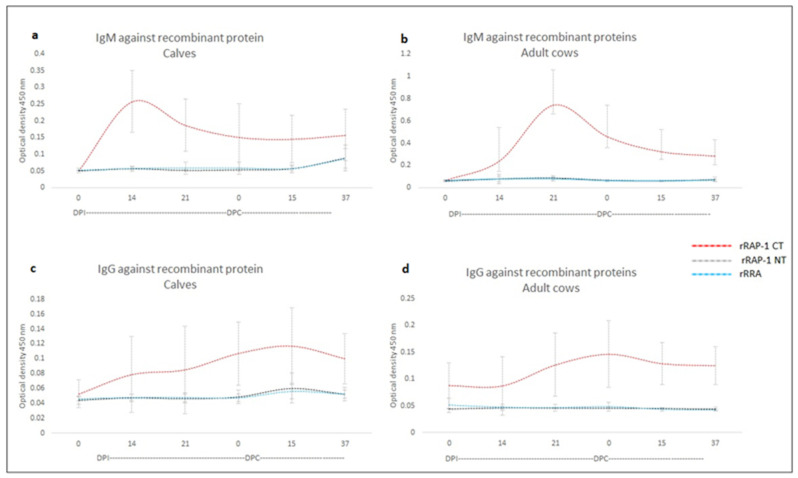
**Sub-immunodominance of Rap-1 NT and RRA proteins.** Recombinant Rap-1 CT, Rap-1 NT, and RRA proteins were exposed to immune sera from cattle vaccinated with attenuated *Babesia bovis* and challenged with a virulent *B. bovis* strain. The binding of antibodies IgM (top panels (**a**,**b**)) and IgG (bottom panels (**c**,**d**)) to the recombinant proteins was evaluated by iELISA. After averaging the Optical Density (OD) values of sera from the calves and the adult cows, rRap-1 CT showed to be an immunodominant antigen, while Rap-1 NT and RRA were immune-subdominant antigens.

The reactivity of rRAP-1 NT, rRRA, and rRAP-1 CT was compared among calves (n = 3) and adult cattle (n = 5) immunized with an in vitro culture-attenuated strain of *B. bovis* (days post-immunization, DPI) and challenged with a virulent *B. bovis* strain (days post-challenge, DPC). Overall, all tested animals developed strong IgM (Supplementary Figure S2A) and IgG (Supplementary Figure S2B) responses against the immunodominant rRAP-1 CT antigen, but significantly lower responses were detected against the rRAP-1 NT and rRRA antigens (ANOVA test *p*-value < 0.05). Interestingly, while all calves developed IgM responses against rRAP-1 CT peaking on day 14 post-vaccination with an attenuated strain, the response is delayed in four out of five adult cows, with the IgM peak appearing 21 days after vaccination (Supplementary Figure S2). On the other hand, both calves and adult cattle developed similar IgM kinetics generating a much weaker peak of IgM responses on day 14. In all cases, the IgM responses detected against rRRA and rRAP-1 NT are significantly weaker compared with rRAP-1 CT (ANOVA test *p*-value < 0.05). No IgM booster responses against the rRAP-1 CT antigen were observed upon challenge with virulent *B. bovis*, but a trend toward increased IgM antibody titers against rRRA seemed to occur after day 15 of the challenge, suggestive of an anamnestic response against this antigen, an outcome that was not evident in adult cows for rRAP-1 NT (Supplementary Figure S2A,B and Figure 1). After averaging the Optical Density (OD) values of sera from the calves C1735, C1736, and C1738, the highest IgM values were rRap-1 CT = 0.2575 ± 0.093, rRap-1 NT = 0.0884 ± 0.038, and rRRA = 0.087 ± 0.030 (Figure 1a), while the highest IgG values were rRap-1 CT = 0.1167 ± 0.051, rRap-1 NT = 0.0603 ± 0.0203, and rRRA = 0.057 ± 0.011 (Figure 1c). Additionally, averaging the OD of sera from the adult cows C1705, C1706, C1707, C1708, and C11325 showed the highest OD values for IgM as follows: rRap-1 CT = 0.739 ± 0.319, rRap-1 NT = 0.0903 ± 0.020, and rRRA = 0.079 (Figure 1b), while the highest OD values for IgG were rRap-1 CT = 0.146 ± 0.062, rRap-1 NT = 0.0458 ± 0.0012, and rRRA = 0.0480 ± 0.0083 (Figure 1d). ANOVA test *p*-value < 0.05 demonstrated significant difference between the level of antibodies against RAP-1 CT compared with the level of antibodies against RAP-1 NT and RRA. Regarding these results, rRap-1 CT showed to be an immunodominant antigen, while Rap-1 NT and RRA can be categorized as sub-immunodominant antigens.

Regarding IgG responses, both calves and adults responded with much higher IgG antibody titers against rRAP-1 CT than against rRRA and rRAP-1 NT. Calves seem to have higher IgG responses than adult cows, a trend suggestive of anamnestic stimulation after the challenge with the *B. bovis* virulent strain, which is not evident in the adult animals. In contrast, the IgG levels against rRAP-1 NT and rRRA are one order of magnitude lower compared to the responses found against rRAP-1CT. IgG anamnestic responses against both rRRA and rRAP-1 NT are also evident in some calves but were not found in adult animals. The average antigenicity of the IgM and IgG responses in calves and adult cattle against rRAP-1 CT, rRAP-1 NT, and rRRA, deduced from these experiments, are also depicted in Supplementary Figure S2A,B. The averaged data presented in Figure 1 confirm the differences in antigenicity among these molecules in immunized and challenged animals and the kinetics in the antibody responses in calves compared to adult cows. However, regardless of the intensity of the immune responses, the data show that RRA and RAP-1 NT contain B-cell epitopes that elicit weak immune responses in immunized cattle.

Taken together, the data mirrored previous observations using synthetic peptides representing immunodominant and sub-immunodominant regions in RAP-1 CT and RRA molecules [11], suggesting that the epitopes present in the RAP-1 CT region of the RAP-1 molecule are highly immunogenic.

**Figure 2:**
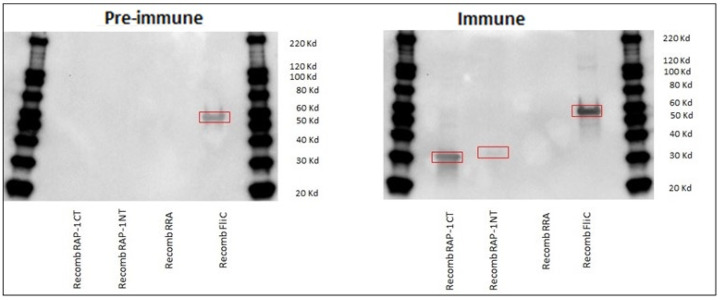
**Western blot anti-recombinant proteins RAP-1 CT, RAP-1 NT, RRA, and FliC.** Pre-immune and immune bovine sera (calf 1735) [1:10] Goat anti-bovine IgG 1:2500). See the corresponding bands inside the red boxes.

In addition, the conclusions derived from the iELISA testing using rRAP-1 NT, rRRA, and rRAP-1 CT were also consistent with the immunoblot analysis shown in Figure 2. Also consistent with the iELISA data is that the sera from a selected immunized and challenged animal (calf 1735) strongly recognized the rRAP-1 CT antigen, but antibodies in this immune serum weakly recognized the rRAP-1 NT antigen in immunoblots. However, no signal was detected with rRRA. Coomassie blue staining and anti-HIS antibody reactivity against all these three recombinant proteins is shown in Supplementary Figure S1. In addition, cattle serum recognized recombinant FliC, which is a component of the adjuvant used in the immunization experiments described below. The immunoblot (Figure 2) revealed that both pre-immune and immune sera from one of the immunized cattle (calf 1735) used in these experiments recognized the rFliC, but, interestingly, the intensity of the signal increased after immunization with attenuated *B. bovis*.

Overall, the data in these experiments confirm RAP-1 NT and RRA as sub-immunodominant antigens, which are appropriate candidates to test whether sub-immunodominant antigens can elicit strong immune responses when administered to cattle with FliC-based adjuvants, as described in the section below.

### 3.2. Immunization of Calves with Recombinant RRA and rRAP-1 NT Using a FliC-Based Adjuvant and Challenge with Virulent Parasites

**Figure 3:**
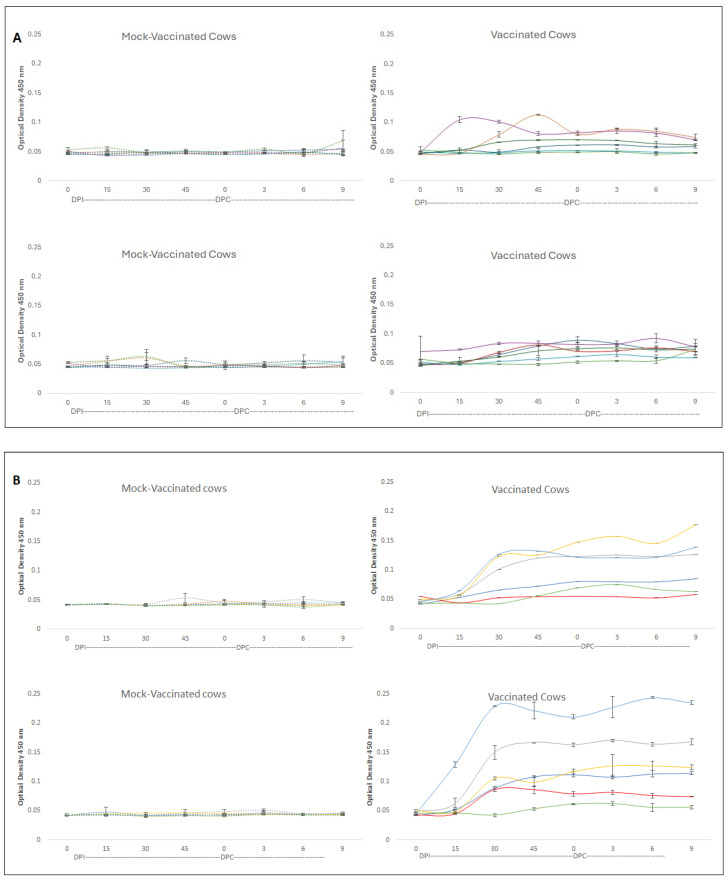
**IgM and IgG reactivity against recombinant RAP-1 NT and RRA.** (**A**) Upper panel: iELISA analysis of IgM antibody levels against rRAP-1 NT in cattle (n = 6) immunized with rRAP-1 NT and RRA using a FliC adjuvant. DPI: days post-immunization with the recombinant antigen mix. DPC represents days post-challenge with the *B. bovis* strain Vir-S74-T3Bo. Lower panel: iELISA analysis of IgM antibody levels against rRRA in cattle (n = 6) immunized with rRAP-1 NT and RRA using a FliC adjuvant. DPI: days post-immunization with the recombinant antigen mix. DPC represents days post-challenge with the *B. bovis* strain Vir-S74-T3Bo. (**B**) Upper panel: iELISA analysis of IgG antibody levels against rRAP-1 NT in cattle (n = 6) immunized with rRAP-1 NT and RRA using a FliC adjuvant. DPI: days post-immunization with the recombinant antigen mix. DPC represents days post-challenge with the *B. bovis* strain Vir-S74-T3Bo. Lower panel: iELISA analysis of IgG antibody levels against rRRA in cattle (n = 6) immunized with rRAP-1 NT and RRA using a FliC adjuvant. DPI: days post-immunization with the recombinant antigen mix. DPC represents days post-challenge with the *B. bovis* strain Vir-S74-T3Bo. Each point represents the average of two replicas, and bars represent the standard deviation. The IDs of the individual animals are as follows: C1 (blue), C2 (red), C3 (grey), C4 (yellow), C5 (cyan), C6 (green), C7 (dotted blue), C8 (dotted orange), C9 (dotted cyan), C10 (dotted yellow), C11 (dotted grey), and C12 (dotted green).

**Figure 4:**
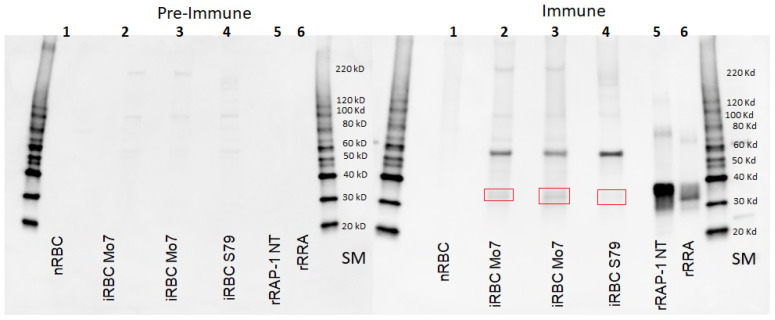
**Immunoblot analysis.** Membranes were incubated with pre-immune and immune bovine sera from the selected cow C1, immunized against rRRA and rRAP-1 NT proteins. Lane 1 represents a lysate from non-infected bovine erythrocytes; Lane 2 represents an Mo7-infected bovine erythrocyte lysate from a 50% parasitemia culture; Lane 3 represents an Mo7-infected bovine erythrocyte lysate from a 65% parasitemia culture; Lane 4 represents lysates derived from the cultured *B. bovis* virulent T2Bo strain used in the challenge; Lane 5 represents recombinant RRA; and Lane 6 represents recombinant RAP-1 NT proteins used in the immunization mix. Red boxes show the corresponding bands. SM: Size marker.

We then tested if immune responses against naturally poor immunogenic antigens such as RAP-1 NT and RRA can be enhanced when the antigens are presented together with a FliC-based adjuvant. To this end, we vaccinated a group of six eight-month calves with a mix of rRRA and rRAP-1 NT in combination with a FliC-based adjuvant. A similar control group of six calves was injected with adjuvant alone. The antibody responses against these antigens are depicted in Figure 3A,B. Both recombinant antigens were able to elicit IgM and IgG immune responses in at least five out of the six immunized animals under the conditions used in the experiment (Figure 3A,B), with calf 6 reacting with a much lower antibody response against both antigens. An immunoblot confirming the specificity of the response of one vaccinated animal (C1) against native *B. bovis* and the recombinant antigens RAP-1 NT and RRA is shown in Figure 4. The antibodies from the vaccinated, but not from the pre-vaccinated calf C1, strongly recognize the recombinant RRA and RAP-1 NT antigens. The statistics for these iELISA results show significant differences among vaccinated and mock-vaccinated animals. A comparison of antigenicity IgM against the rRRA-vaccinated vs. mock-vaccinated *t*-test = 0.00149, and a comparison of antigenicity IgM against the rRAP-1 NT-vaccinated vs. mock-vaccinated *t*-test = 0.00182. Also, a comparison of antigenicity IgG against the rRRA-vaccinated vs. mock-vaccinated *t*-test = 0.00167, and a comparison of antigenicity IgG against the rRAP-1 NT-vaccinated vs. mock-vaccinated *t*-test = 0.00152. In addition, the antibodies in the immunized calf C1 also recognize a native protein of ~60 kDa (presumably native RAP-1) and ~38 kDa (presumably native RRA, red box). These patterns of reactivity contrast sharply with the reactivity of antibodies in calves vaccinated with attenuated parasites and challenged with virulent parasites, shown in Figure 1, evidencing the increase in immunogenicity of subimmunodominant antigens upon vaccination with recombinant antigens and a FliC-based adjuvant.

**Figure 5:**
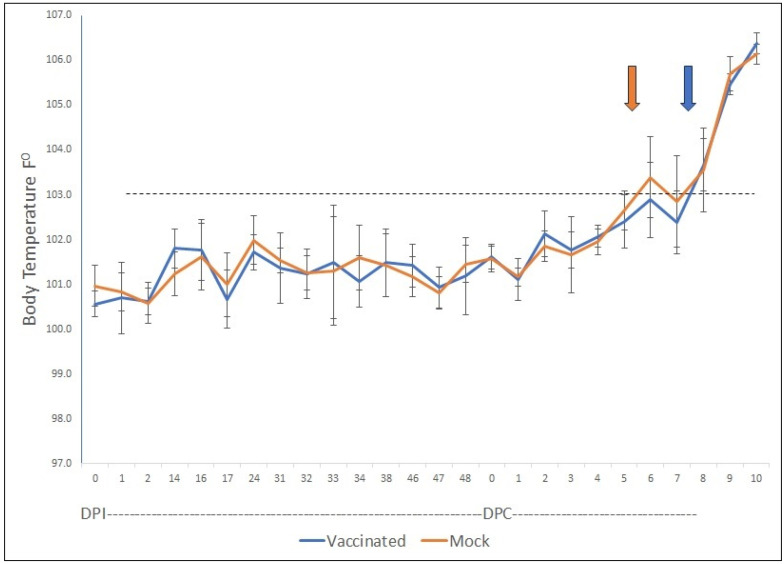
**Body Temperature.** Averaged body temperature for the six vaccinated cows and the six mock-vaccinated cows during the days post-immunization (DPI) with the rRRA and rRap-1 NT antigen mix and the days post-challenge (DPC) with a virulent *B. bovis* strain. Orange and blue arrows indicate the onset of critical temps (>103 °F).

The challenge with virulent parasites performed two weeks after the last inoculations resulted in acute infections in both groups of animals. However, a closer analysis of the resulting clinical and analytical data demonstrated interesting differences between the two groups in some of the parameters measured. First, although the average increase in rectal temperature (RT) was indistinguishable between the two groups of animals (Figure 5), the non-vaccinated group (average values) reached the critical value of RT, considered as an elevated fever (103 °F), two days before the vaccinated group (Figure 5).

**Figure 6:**
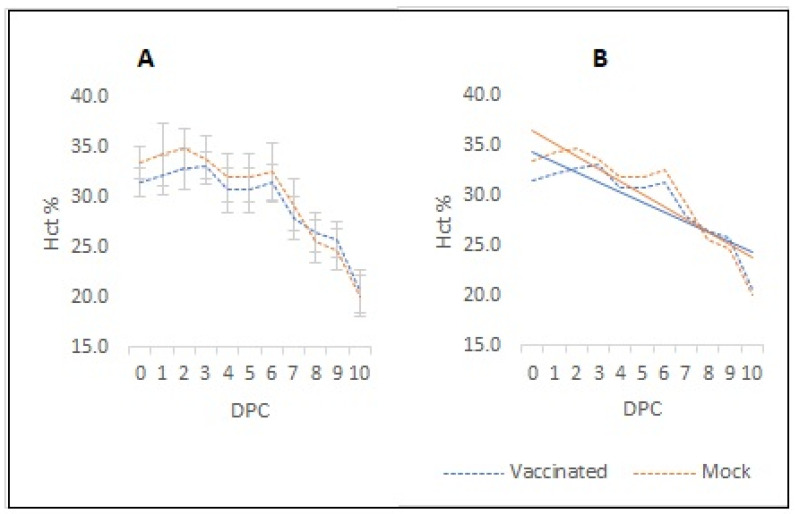
**Hematocrit.** Averaged hematocrit values for the six vaccinated cows and for the six mock-vaccinated cows during days post-challenge (DPC) with a virulent *B. bovis* strain (**A**). Slope lines for the averaged hematocrit from the six vaccinated (blue line) and six mock-vaccinated (orange line) cows (**B**).

In addition, the average rate of Hct decrease is different among the vaccinated and mock-vaccinated groups (Figure 6). We calculated the hematocrit decay slopes for each group applying the formula described in Section 2: The calculated slope value for the mock-vaccinated group was −1.2771, and for the vaccinated group, it was −0.9891, implying a slower rate of Hct decay. Accordingly, the calculated days required to reach 50% of the initial average Hct% values for the vaccinated group were 17.9 and for the mock-vaccinated group it was 14.8 DPC. It is possible that these differences may be due to an antibody-mediated parasite neutralization effect in vaccinated animals.

**Figure 7:**
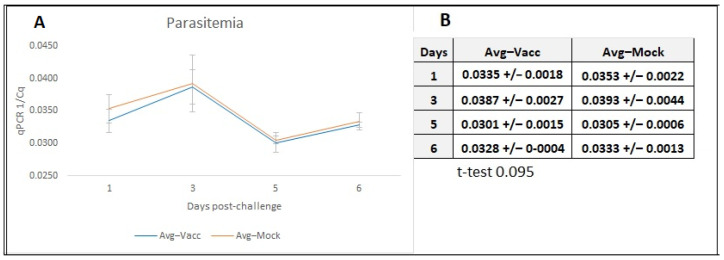
**Parasite load.** Estimation of parasite loads using qPCR: Quantification Cycle (Cq) values were obtained using a quantitative PCR (qPCR) analysis, performed on total DNA in blood from the six vaccinated and the six mock-vaccinated cows during days post-challenge (DPC). (**A**) Graphical representation. The 1/Cq value was plotted, since the Cq values represent values that are inverse to parasite load. (**B**) Table describing the qPCR numerical data. Estimation of the average 1/Cq values obtained from the qPCR analysis on samples from vaccinated and mock-vaccinated cows. This analysis was performed on days 1, 3, 5, and 6 post-challenge. Significative differences for day 1 (*p* < 0.095) were determined using the *t*-test.

We also quantified parasitemia by qPCR. The Quantification Cycle (Cq) of the qPCR is an indirect measurement of parasitemia. Since the Cq values are inverse to the parasitemia, the reverse of this figure, 1/Cq, was plotted in the figure to facilitate a more intuitive interpretation of the data (Figure 7). Comparisons of the Cq average values determined on the blood samples from the vaccinated and the mock-vaccinated animals showed a lack of statistical difference between the two groups (*p* = 0.095). However, the parasitemia average of the vaccinated cows was lower than the mock-vaccinated animals in all days tested. Additionally, the parasitemia for the DPC 1 was significant lower for four out of the six vaccinated animals (C1, C2, C3, and C6) (29.94 ± 1.54) than for the mock-vaccinated animals (28.42 ± 1.74) (*p* = 0.03).

**Figure 8:**
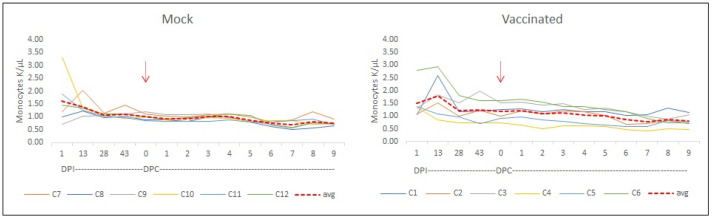
**Monocytes.** Blood cell count in mock-vaccinated and rRRA–rRAP-1 NT-vaccinated cows. Monocytes: The analysis was performed during the vaccination phase and after the challenge showed an increase in absolute number of monocytes in 3 out of 6 vaccinated animals. Comparison between the monocyte count average from the mock-vaccinated cows and each one of the six vaccinated animals showed an increase in these values for cows C1, C3, and C6 (*p* < 0.05). Challenge day (red arrow).

Blood cell counts were also performed before and after the challenge in all experimental animals. Monocyte counts, during the vaccination and after the challenge, showed an increase in absolute number in three out of six vaccinated animals (Figure 8). A comparison between the monocyte count average from the mock-vaccinated cows and each one of the six vaccinated animals showed an increase in these values for cows C1, C3, and C6 (*p* < 0.05).

**Figure 9:**
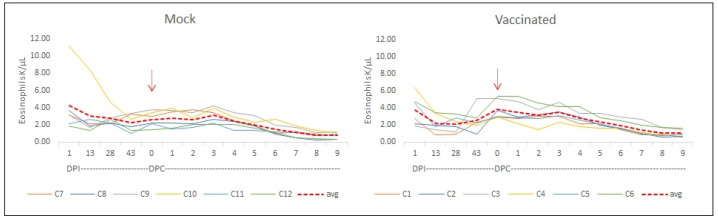
**Neutrophils.** Blood cells count in mock-vaccinated and rRRA–rRAP-1 NT-vaccinated cows. Neutrophils: The analysis was performed during the vaccination phase and after the challenge showed an increase in absolute number of monocytes in 3 out of 6 vaccinated animals. Comparison between the neutrophil count average from the mock-vaccinated cows and each one of the six vaccinated animals showed an increase in these values for cows C3 and C6 (*p* < 0.05). Challenge day (red arrow).

Neutrophil counts during the vaccination and after the challenge showed an increase in absolute number in two out of six vaccinated animals (Figure 9). Comparison between the neutrophil count average from the mock-vaccinated cows and each one of the six vaccinated animals showed a significant increase in these values for vaccinated cows C3 and C6 (*p* < 0.05). Lymphocyte counts during the vaccination and after the challenge showed no increase in absolute number between vaccinated and mock-vaccinated animals (Supplementary Figure S3). Furthermore, a comparison between the lymphocyte count average from the mock-vaccinated cows and each one of the six vaccinated animals showed no increase in these values.

The trial was finished on day 13 post-challenge, and all animals were humanely euthanized according to ethical animal care requirements, so we were not able to verify the hematocrit decay predictions or determine if any of the experimental animals would survive the challenge without pharmacological intervention.

## 4. Discussion

In this study, we first characterized the dynamics of the antibody responses against recombinant proteins representing immune-subimmunodominant antigens (RRA and RAP-1 NT) and the known immunodominant antigen RAP-1 CT. To this end, we compared levels of antibodies against RAP-1 NT, RRA, and RAP-1 CT in cattle vaccinated with a live *B. bovis* vaccine and challenged with virulent strain of *B. bovis*. Once the sub-immunodominance of RRA and RAP-1 NT was demonstrated by serological analysis, we used these two antigens in conjunction with the FliC–Emulsigen mix adjuvant to immunize a group of cattle. At the time of this study, FliC-based adjuvants were untested for *Babesia* vaccine development despite the apparent advantages that this compound can offer, which include increased antibody and cellular responses of the Th I type [17]. We demonstrated hereby that cattle immunized with a combination of two related recombinant antigens representing the sub-immunodominant RAP-1 NT portion of RAP-1 and RRA using a FliC-containing adjuvant elicit high levels of antibody responses in at least five of the six animals in the vaccinated group, with one cow (C6) displaying lower antibody responses against both antigens, when compared to the other five immunized animals. We do not have a definitive explanation for the lack of response of this animal, but it may be due to a different Mayor Histocompatibility Complex MHC composition, unknown previous history of infectious or autoimmune disease, or other undetermined factors. Regardless of this, these are very interesting observations and lessons derived from the challenge experiment that can help guide future research aimed at developing subunit vaccines against *B. bovis*.

Several lines of evidence suggest that the vaccination of cattle with a recombinant RRA/RAP-1 NT mix with a FliC-derived adjuvant had a potentially protective effect upon the challenge with a virulent strain of *B. bovis*. Therefore, despite the lack of overall statistically significant differences between the two groups, several post-challenge parameters measured in this study are different and likely biologically significant, when comparing both immunized and mock-immunized groups, involving at least 50% of the animals in the vaccinated group. Firstly, there was a difference in the slope of the average drop in hematocrit between both groups. The significance of this observation is two-fold. There are individual variances in the rate of hematocrit decay, which in turn results in differences in the slopes deduced from the two groups. Secondly, using the calculated slopes, the data allow for a prediction of the average day when the animals may reach a critical value in their hematocrit. Such a critical point would be typically reached when a 50% decrease in hematocrit occurs as a result of the infection. Thus, whereas the unvaccinated group would reach a 50% decrease in the hematocrit, at least on average, by day 15 post-challenge, the vaccinated group would reach it at 18 days post-challenge. Although this is a mathematical estimation, this difference in the time required for a critical hematocrit drop might be enough, at least in theory, for the vaccinated animals to mount a protective immune response and thus ultimately survive the challenge. We were unable to determine if that was the case in this experiment since we had to interrupt the trial and proceed to euthanasia of all animals earlier than those days due to humane reasons. Importantly, four calves (C1, C2, C3, and C5) displayed slopes in their hematocrit decrease curves that are lower than their group average, a finding that may have other interesting implications, as discussed below. Secondly, there are individual differences in the number of parasites in circulation in the challenged animals; again, this involves animals C1, C2, C3, and C6 with values that are lower than the group average. In addition, the initial average number of circulating parasites is significantly lower on day 1 post-challenge in the vaccinated group. Thirdly, an early monocyte increase was recently identified as a correlate of protection against acute *B. bovis* infection [19]. We also found a differential response in monocyte counts upon the challenge in three out of the six vaccinated animals (C1, C3, and C6). These increases were not observed in any animal of the control group. In addition, a trend of an increase in the number of neutrophils was also found for vaccinated calves C3 and C6.

Considering that RRA (and perhaps RAP-1 NT) contains neutralization-sensitive epitopes, it is possible to speculate that the antibody responses against these antigens may be responsible for the initial reduction in the number of circulating parasites. Afterward, it is possible that animals became overwhelmed by the fast increase in the number of circulating parasites, and the ensuing depletion of neutralizing antibodies, starting on day 2 after the challenge, when the number of parasites circulating in these animals is seen at the same level as those in the control group. Interestingly, and consistent with this postulation, animal C6, which showed the lowest antibody response to immunization, is among the animals without reduced parasitemia on day 1 post-challenge. Nevertheless, C6 did show some signs of a protective immune responses upon vaccination, perhaps due to the activation of cellular immunity as suggested by an increase in the number in monocytes and neutrophils. Also, it is possible to speculate that this subtle effect might be related to the use of FliC as an adjuvant. It would be important to perform further experiments using distinct amounts and formulations of the adjuvant mix containing FliC and to verify if such changes can influence antibody levels and, ultimately, the outcome of the challenge. Interestingly, vaccinated cow 3 shows consistent positive protection-like data in all parameters tested.

In addition to the differential responses among animals in the vaccinated group discussed here, we also found a difference between groups regarding the average starting day for the appearance of critical body temperatures (above 103 °F) for the non-vaccinated group, which may also be related to the differences in responses detailed above.

Finally, we would like to add that the challenge model used in this study is highly demanding for the cattle hosts and may not likely be realistic, nor representative, of natural *Babesia* infections. To this end, it must be considered that in nature, cattle are usually infected not through the merozoite but the sporozoite stage of *B. bovis* inoculated via *B. bovis*-infected tick infestation. It can be realistically expected that such a natural infection challenge may result in completely different results in vaccine trials, since several variables, including parasite dose, parasite infective stage (sporozoite vs. merozoite), and the presence of other immunomodulant and anticoagulant factors included in the tick saliva may play important roles that may affect the outcome of the challenge. However, although more realistic, such a *Babesia*-infected tick challenge is difficult to standardize, but a more natural challenge would be possible using a known dose of free sporozoites, if these can be isolated from salivary glands of the ticks. We thus propose that the development of new and more realistic challenge models is imperative to further evaluate protective immune responses upon immunization, which will be required for developing subunit vaccines against bovine babesiosis.

In summary, this study demonstrated the elicitation of antibody responses against RRA and RAP-1 NT, two poorly antigenic, sub-immunodominant antigens of *B. bovis*, adjuvanted with FliC. This attribute may be important to test the concept of using sub-immunodominant antigens as effective vaccine components. We also showed individual cattle responses to vaccination that are indicative of protective immune responses, or at least that are distinct than those in the mock-vaccinated animals. The delay in the hematocrit decrease, as shown by its slope rate, is an interesting novel finding and is a parameter that could be included in the evaluation of future vaccine trials. Finally, altogether with numerous previous similar *Babesia* vaccine testing studies, the data strongly suggest the need to develop a more realistic challenge model for future evaluation of vaccine candidate antigens against *Babesia* parasites, which is not based on the inoculation of large amounts of highly virulent merozoites, such as a sporozoite-based model. Overall, we believe that the data emerging from this study are promising for the development of subunit vaccines against *B. bovis*, especially if more appropriate challenge models are developed, more protective antigens are identified, and the nature of protective immune responses can be better elucidated.

Future research with larger sample sizes and extended observation periods will be needed to fully optimize the vaccine’s protective capabilities. In addition, future work will be needed on developing a more realistic challenge model for *Babesia* and to test promising combinations of sub-immunodominant and immunodominant recombinant vaccine candidate antigens using FliC adjuvants against these devastating parasites.

## 5. Conclusions

The data gathered in this study provide confirmation of the sub-immunodominant antigenicity of RRA and the RAP-1 NT portion of RAP-1 and identified an effective method for increasing the immunogenicity of ISD antigens based on a novel FliC-based adjuvant approach. The vaccination of cattle with RRA and RAP-1 NT with a FliC-based adjuvant generated an immune response that decreases the rate of decay of hematocrit and elicited earlier monocyte responses in at least half of the vaccinated animals. Overall, the study provides valuable insights for the future development of subunit vaccines that are effective against *B. bovis*.

## Data Availability

Data are available in the current manuscript.

## Supplementary Materials

The following supporting information can be downloaded at https://www.mdpi.com/article/10.3390/vaccines12111215/s1, Figure S1: Western blot anti-HIS. *Escherichia coli* recombinant proteins: Rap-1 CT, Rap-1 NT, RRA, and FliC and Coomassie blue staining of the purified recombinant RAP-1CT, RAP-1 NT, and RRA proteins separated on an SDS-PAGE gel; Figure S2: Recombinant protein average antigenicity IgM and IgG; Figure S3: Lymphocyte counts on vaccinated and mock animals.
