# Supplementary material for: Evaluation of the Use of Sub-Immunodominant Antigens of Babesia bovis with Flagellin C Adjuvant in Subunit Vaccine Development
Source: Vaccines (Basel). 2024 Oct 25;12(11):1215. doi: 10.3390/vaccines12111215 (PMC11598123; doi:10.3390/vaccines12111215)
Supplement: Supplementary file 1 [file vaccines-12-01215-s001.zip › vaccines-3190715-supplementary.pdf]

vaccines MDPI

Supplementary Figures

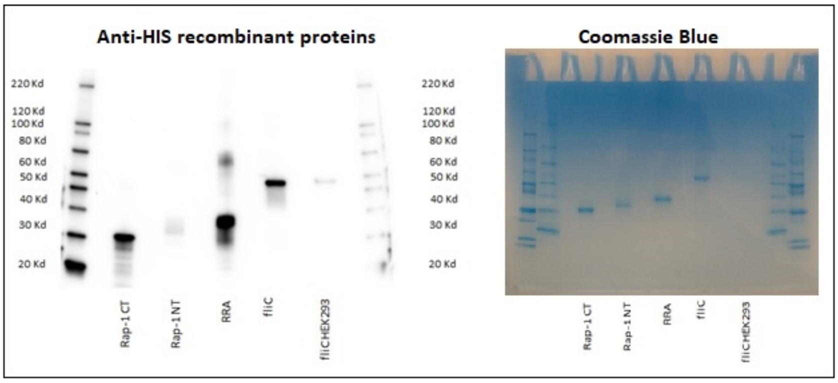

Supplementary Figure S1. Left Panel: Western blot anti-HIS. *E. coli* recombinant proteins: Rap-1 CT, Rap-1 NT, RRA, and FliC. 1.25 ug from each protein. Primary Ab anti-HIS HRP conjugated [1:500]. **Right Panel:** Coomassie blue staining of the purified recombinant RAP-1CT, RAP-1NT and RRA proteins separated on a SDS-PAGE gel

A

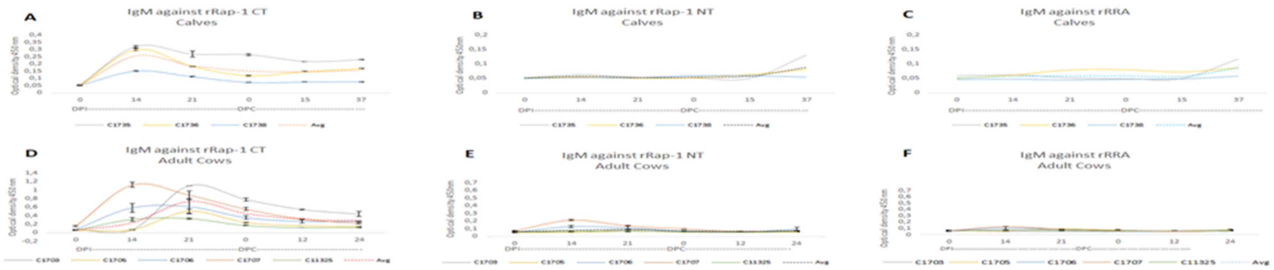

B

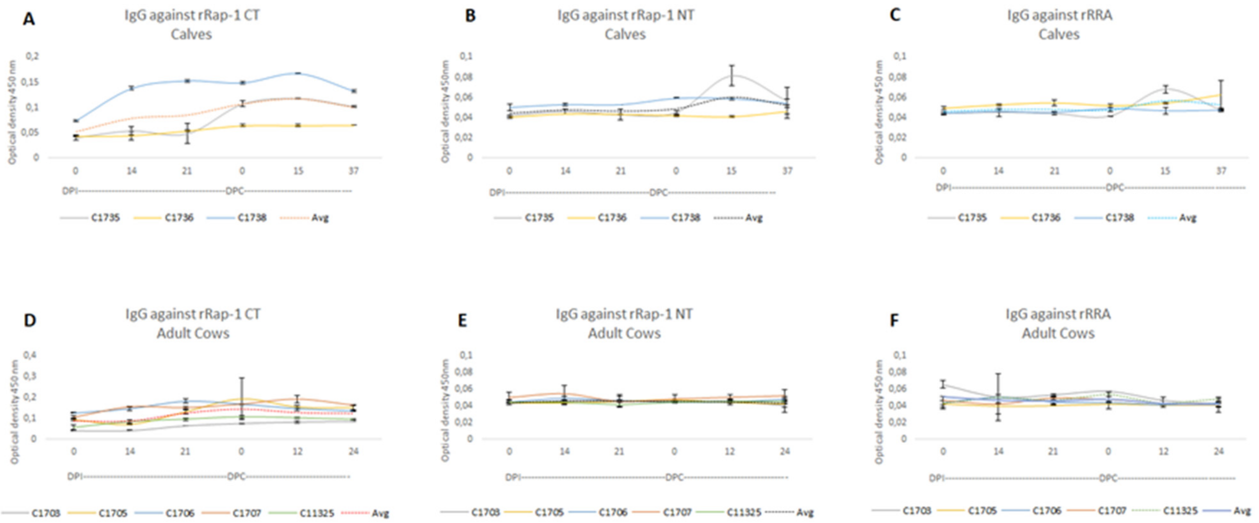

Supplementary Figure S2. IgM and IgG reactivity against recombinant RRA, RAP-1NT and RAP-1 CT proteins. *Panel A*: iELISA analysis of IgM antibody levels against RAP1 CT, RAP1 NT and RRA recombinant proteins in cattle experimentally immunized and challenged with attenuated and virulent *B. bovis* respectively. (a) IgM levels in calves against rRAP-1CT; (b) IgM levels in calves against rRAP1 NT. (c) IgM levels in calves against rRRA; (d) IgM levels in adult cattle against rRAP-1 CT. (e) IgM levels in adult cattle against rRAP 1NT; (f) IgM levels in adult cattle against rRRA. *Panel B*: iELISA analysis of IgG antibody levels against RAP1 CT, RAP1 NT and RRA recombinant proteins in cattle experimentally infected and challenged with attenuated and virulent *B. bovis* respectively. (a) IgG levels in calves against rRAP-1CT; (b) IgG levels in calves against rRAP1 NT. (c) IgG levels in calves against rRRA; (d) IgG levels in adult cattle against rRAP-1 CT. (e) IgG levels in adult cattle against rRAP 1NT ; (f) IgG levels in adult cattle against rRRA. In both panels A, and B; DPI: days post-infection with the *B. bovis* attenuated strain Att- S74-T3Bo. DPC: represents days post challenge with the *B. bovis* strain Vir-S74-T3Bo. The IDs of the calves tested are as follows: C1738, C1736 C1735. The IDs of the adult cattle tested are as follows: C1703, C1707, C11325, C1706, C1707. Dotted lines represent the average of all ODs for each date for all animals in each group.

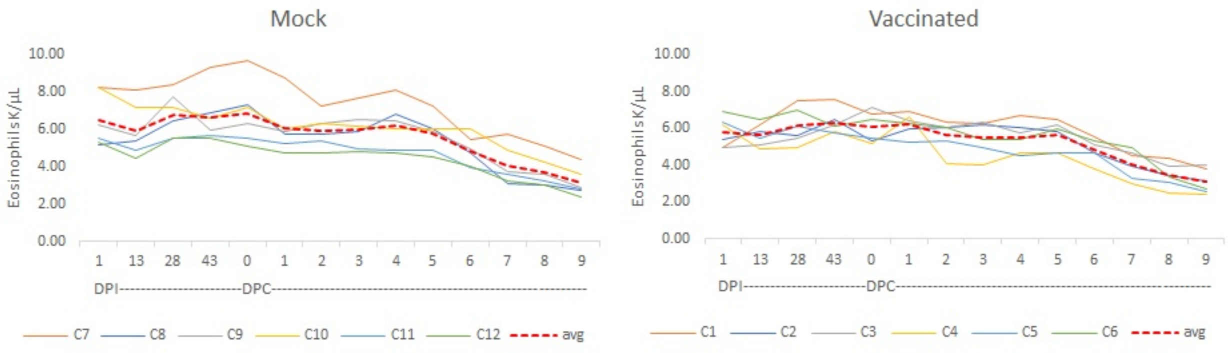

Supplementary Figure S3. Blood lymphocyte counts were performed on animals of both experimental groups. Blood cell count was performed during the vaccination phase (DPI) and after the challenge (DPC) (Day 0). The data showed no increase in absolute number of lymphocytes between vaccinated and mock-vaccinated animals.
